# Intra- and inter-operator reliability of three-dimensional preoperative planning in total knee arthroplasty

**DOI:** 10.1007/s00402-024-05438-8

**Published:** 2024-07-15

**Authors:** Killian Cosendey, Kevin Moerenhout, Julien Stanovici, Brigitte M. Jolles, Julien Favre

**Affiliations:** 1 Department of Musculoskeletal Medicine (DAL), Lausanne University Hospital and University of Lausanne (CHUV-UNIL), Avenue Pierre-Decker 4, Lausanne, CH-1005 Switzerland; 2 Institute of Electrical and Micro Engineering, Ecole Polytechnique Fédérale de Lausanne (EPFL), Lausanne, Switzerland; 3 The Sense Innovation and Research Center, Lausanne & Sion, Switzerland

**Keywords:** Total knee arthroplasty, Preoperative planning, Reliability, Implant size selection, Implant placement

## Abstract

**Purpose:**

To characterize the intra- and inter-operator reliability of a CT-based 3D preoperative planning software.

**Materials and methods:**

This study analyzed 30 CT scans of de-identified knees with osteoarthritis. For each scan, a case planner segmented the bones and pre-planned the TKA. Three orthopedic surgeons then reviewed each pre-planning three times at least one week apart, in a blinded manner. During the reviews, the surgeons modified the pre-plannings until they felt the plannings matched the objectives defined collegially at the beginning of the study. Reliability was assessed using the Intraclass Correlation Coefficient (ICC) and the Standard Error of Measurement (SEM).

**Results:**

The intra- and inter-operator reliabilities for implant size selection were almost perfect (ICC between 0.97 and 0.99). Implants of same sizes were selected in 67.1–90.0% of cases. For implant placements, almost perfect intra- and inter-operator reliability was observed in all degrees-of-freedom (ICC between 0.81 and 1.00), except in flexion-extension for the femur (intra-operator ICC between: 0.76 and 0.99; inter-operator ICC of 0.61) and the tibia (intra-operator ICC between 0.12 and 1.00; inter-operator ICC of 0.03). All implant placements SEM were below 1.3 mm or 1.7°.

**Conclusions:**

This study showed high intra- and inter-operator reliability for implant size selection and, in most of the degrees-of-freedom, also for implant placements. Further research is needed to evaluate the benefit of developing more precise means of describing the objectives of the surgical planning as well as to evaluate the possibility and relevance of adding features in the planning software to assist the operators.

## Introduction

Total Knee Arthroplasty (TKA) has become a common orthopedic procedure, with about 680’000 primary TKA surgeries performed each year in the United States [1]. Despite this large number, up to 18% of patients are unsatisfied with the function of their knee after TKA [2, 3]. Literature has related inappropriate size, malalignment, and misplacement of the implants to poorer outcomes [4, 5]. These findings highlight the importance of considering these factors carefully. They also encourage the development of means to improve their management.

Preoperative planning combined with assistive devices, such as robotic-assisted systems, computer navigations or patient-specific instrumentations, provides an opportunity to improve implant size selection, bone resection and implant placements [5–9]. In general, preoperative planning includes two phases: (1) an image acquisition phase, typically using magnetic resonance imaging (MRI) or computed tomography (CT) to determine the three-dimensional (3D) geometry of the knees and (2) a planning phase, consisting in segmenting the images to reconstruct 3D models of the patients’ bones, identifying bony landmarks, selecting the prosthesis model and size, and virtually placing the prosthesis on the 3D bone models. While preoperative planning is used worldwide, there has been limited research on this procedure.

The reliability of the implant size selection and of the implant placements in the planning software remain poorly documented. So far, one study analyzed the intra- and inter-operator reliability when planning primary TKA based on CT scan. It reported an excellent reliability for implant size (ICC range from 0.91 to 0.94), but moderate reliability for implant orientation (ICC range from 0.47 to 0.89) [10]. Another study using MRI for preoperative patient-specific instrumentation (PSI) planning observed an almost perfect intra- and inter-operator reliability for implant size (ICC range from 0.91 to 0.99), but the results were more heterogeneous for implant position (ICC range from 0.18 to 1.00) and orientation (ICC range from 0.08 to 1.00) [11]. These prior studies provide an interesting estimation. However, since many elements could influence the reliability results, such as the planning software, the surgical objectives, or the operators, there is a need to complete these prior works. The TSolution One Surgical System^®^ (Think Surgical Inc., California, USA) includes a surgical planning workstation (named TPLAN^®^) that would be interesting to assess for itself and to consolidate the field. Indeed, on one hand, evaluating this other option could confirm the reliability ranges previously published and therefore suggest that reliability is rather universal. On the other hand, it could indicate variations in reliability data and possibly highlight elements that could affect the reliability.

The objectives of the study were to characterize the intra- and inter-operator reliability of the TPLAN^®^ surgical planning workstation for implant size selection and implant placements.

## Materials and methods

Three orthopedic surgeons with prior experience in 3D surgical planning participated in this IRB-approved study after completing the standard training dispensed by the planning software manufacturer. At the beginning of the study, the surgeons agreed on the surgical planning objectives, which were to have a neutral mechanical axis, a femoral axial rotation based on the transepicondylar axis and a tibial slope restoring the bone morphology with a cap at 5°. This study used exclusively “Unity Posterior Stabilized Femoral” and “Unity Tibial” implants (Corin, Cirencester, UK).

Thirty CT scans of de-identified knees with osteoarthritis were uploaded and processed in TPLAN^®^. A sample size of 30 knees was chosen, according to the statistical methods described by Walter et al. [12], to detect expected reliability of 0.8 (ρ_0_), considering a minimal level of reliability of 0.6 (ρ_1_), α of 0.05 and β of 0.2. For each knee, a case planner from Think Surgical Inc. segmented the bones and pre-planned the TKA using TPLAN^®^ according to the planning objectives. The pre-planning consisted in identifying bony landmarks, selecting implant sizes, and placing the implants relative to the 3D bone models. The surgical planning workstation provided assistance for achieving certain objectives, such as positioning the implant in a neutral mechanical axis and parallel to the transepicondylar axis, through coronal and transverse alignment presets.

**Fig. 1:**
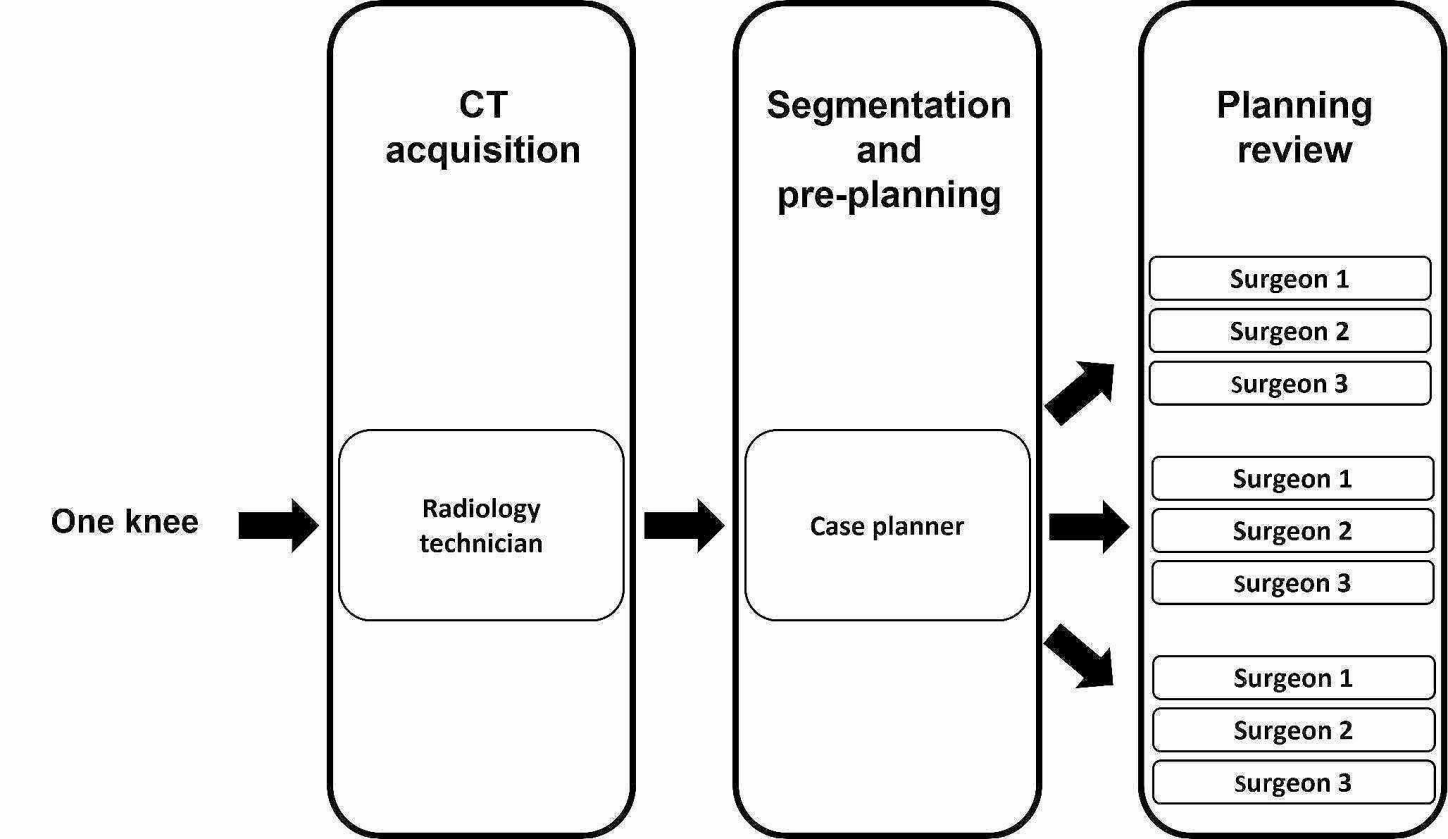
Study chart for one knee (30 knees in total)

After the pre-planning, each surgeon reviewed the 30 cases three times (Fig. 1). The orders in which the surgeons reviewed their 90 cases were randomized, and there was a minimum of one week between reviews of the same knee. The surgeons were provided with a series of 90 cases without indication of which cases were from the same knees. In addition, no feedback was provided to the surgeons during the review of the 90 cases. The review process included two steps. First, the surgeons determined the implant sizes they thought would be the most adapted, independently of the sizes selected during the pre-planning and reported these sizes in a chart. Then, they examined and, if necessary, modified the pre-planning according to the planning objectives. For this, the surgeons could modify the identification of the bony landmarks and the position and orientation of the implants, but not the implant sizes. The use of predetermined implant sizes excluded the possibility of having knees planned with different implant sizes which would have biased the analysis of the implant position and orientation reliability.

### Reliability analysis

The implant position and orientation were described with respect to the anatomical frames [13]. The position was measured along the anterior-posterior, proximal-distal and medial-lateral axes and the orientation using angles in the sagittal, frontal and transverse planes. The reliability was not assessed for the femur and tibia in the coronal plane and for the femur in the transverse plane because these degrees-of-freedom were not at the discretion of the surgeons but fixed by the planning objectives and forced by the planning software.

The reliability in implant sizes and implant placements were described with Intraclass Correlation Coefficient (ICC) and Standard Error of Measurement (SEM). ICC was used to estimate the degree of reliability, while SEM was reported to quantify the magnitude of variations. The intra-operator reliability was evaluated independently for each surgeon and degree-of-freedom based on the three measures for the 30 knees. For the inter-operator reliability, a bootstrap method was used consisting of random selections of one measure for each surgeon (out of the three). The interpretation guidelines of Landis and Koch were used, with ICC between 0.0 and 0.2 indicating slight reliability, between 0.21 and 0.4 indicating fair reliability, between 0.41 and 0.6 indicating moderate reliability, between 0.61 and 0.8 indicating substantial reliability, and between 0.81 and 1.0 indicating almost perfect reliability [14]. For the implant size selection, the reliability was also assessed using the percentage agreement for exact size, as well as for each size unit of disagreement. Data processing and statistical analysis were done with Matlab (Mathworks, Natick, MA).

## Results

Analyzing the surgeons individually indicated that they selected the same implant size 76.7–90.0% of the time for the femur and 73.3–83.3% of the time for the tibia. When considering a one size difference, the percentage increased to 100% for the three surgeons and both bones, meaning that the size selection never differed by more than one unit. The results were similar inter-operator, with a selection of the same implant size 79.6% of the time for the femur and 67.1% of the time for the tibia, and an increase to 100% when considering one size difference. All the implant size ICC were considered almost perfect (between 0.97 and 0.99), with SEM between 0.15 and 0.3 unit (Table 1).

**Table 1:** Intra- and inter-operator reliability (*n* = 30 knees)

		Femur				Tibia				
		Intra-opertor Surgeon 1	Intra-opertor Surgeon 2	Intra-opertor Surgeon 3	Inter-opertator	Intra-opertor Surgeon 1		Intra-opertor Surgeon 2	Intra-opertor Surgeon 3	Inter-opertator
Implant size	ICC (CI)	0.99 (0.98–0.99)	0.97 (0.95–0.99)	0.99 (0.99-1.00)	0.98 (0.98–0.98)	0.98 (0.97–0.99)		0.98 (0.96–0.99)	0.99 (0.98–0.99)	0.98 (0.98–0.98)
	SEM, size unit	0.18	0.28	0.15	0.25	0.26		0.30	0.21	0.30
Position										
Anterior-posterior	ICC (CI)	1.00 (0.99-1.00)	0.90 (0.81–0.95)	0.98 (0.95–0.99)	0.86 (0.85–0.86)	0.98 (0.96–0.99)		0.94 (0.89–0.98)	0.97 (0.94–0.99)	0.93 (0.93–0.93)
	SEM, mm	0.26	0.90	0.57	1.40	0.37		0.59	0.42	0.59
Proximal-distal	ICC (CI)	0.99 (0.98-1.00)	0.84 (0.71–0.92)	0.93 (0.86–0.97)	0.85 (0.85–0.86)	0.99 (0.98–0.99)		0.87 (0.75–0.94)	1.00 (1.00–1.00)	0.92 (0.92–0.92)
	SEM, mm	0.15	0.87	0.44	0.68	0.14		0.44	0.00	0.42
Lateral-medial	ICC (CI)	0.92 (0.84–0.96)	0.84 (0.71–0.92)	0.81 (0.67–0.91)	0.82 (0.82–0.82)	0.98 (0.95–0.99)		0.96 (0.92–0.98)	1.00 (0.99-1.00)	0.95 (0.95–0.95)
	SEM, mm	0.41	0.57	0.65	0.56	0.39		0.38	0.12	0.40
Orientation										
Transverse plane	ICC (CI)	n/a	n/a	n/a	n/a	0.98 (0.95–0.99)		0.91 (0.81–0.96)	0.98 (0.95–0.99)	0.91 (0.91–0.91)
	SEM, °	n/a	n/a	n/a	n/a	1.12		1.59	0.90	1.74
Sagittal plane	ICC (CI)	0.99 (0.97–0.99)	0.76 (0.59–0.88)	0.94 (0.88–0.97)	0.60 (0.60–0.60)	0.44 (0.20–0.67)		0.12 (-0.13-0.44)	1.00 (1.00–1.00)	0.03 (0.03–0.03)
	SEM, °	0.18	1.23	0.36	1.25	0.81		1.49	0.01	1.73

ICC: Interclass correlation coefficient; CI: 95% Confidence interval; SEM: Standard error of measurement

The intra-operator reliability for the femur and tibia were almost perfect (ICC between 0.81 and 1.00), except in the femoral sagittal plane, where it was substantial for one surgeon (ICC of 0.76), and in the tibial sagittal plane, where it was moderate (ICC of 0.44) and slight (ICC of 0.12) for two surgeons. The intra-operator SEM were below 1 mm or 1°, except for one surgeon in the femoral sagittal plane (SEM of 1.23°), tibial transverse plane (1.59°) and tibial sagittal plane (1.49°) and for another surgeon in the tibial transverse plane (1.12°).

The inter-operator reliability for the femur and tibia were almost perfect (ICC between 0.83 and 0.99), except for the femoral sagittal plane, where it was moderate (ICC of 0.60), and the tibial sagittal plane, where it was slight (ICC of 0.03). The inter-operator SEM were below 1 mm or 1°, except for the femur along the anterior-posterior axis and in the sagittal plane (SEM of 1.40 mm and 1.25°, respectively) and for the tibia in the transverse and sagittal planes (SEM of 1.74° and 1.73°, respectively).

## Discussion

This study showed a high level of agreement in implant size selection, with the same size selection at least 67% of the time and differences never exceeding one size. These results are in agreement with prior studies using other planning methods, where same size selection was reported to vary between 44.3% and 66.7% [10] and ICC ranged from 0.84 to 0.99 [11]. Altogether, this is encouraging as inappropriate implant size has been related to various complications and inferior clinical outcomes. Specifically, oversizing of the femoral implants has been associated with increase in pain, decrease in knee function and lower postoperative flexion [15, 16]. Regarding the tibial implants, oversizing has been associated with higher risks of inferior knee injury, increased pain and inferior functional outcomes [15, 17, 18], while undersizing has been related to an increased rate of tibial implant loosening and prosthetic failure [19]. The results are also encouraging regarding costs and operating room efficiency as several studies have reported larger burden when the differences in implant sizes varies by more than one size between the planning and the surgery [20, 21].

Both intra- and inter-operator implant placements demonstrated almost perfect reliability (ICC above 0.81) and low variation among plannings (SEM below 1 mm or 1°) for all degrees-of-freedom, except for four that are discussed hereafter. Along the femoral anterior-posterior axis, the ICC was lower and the SEM larger among surgeons than within surgeons, suggesting that the surgeons remained consistent over time, but understood the implant placements objectives differently. In the tibial transverse plane and in the femoral and tibial sagittal planes, the reliability was lower both intra- and inter-operator, suggesting less specific definitions for the placements of the implants in these degrees-of-freedom. These observations question the possibility and relevance of adding new features in planning software to assist the operator. Such additions could indeed improve the reliability particularly in the four degrees-of-freedom mentioned above. This being said, it is important to note that the variations among plannings remained relatively low both intra- and inter-operator, with SEM values below 1.3 mm or 1.7°. Consequently, to push the interpretation further and spot effective sources of improvement, additional research on the clinical impact of the planning reliability appears necessary.

Compared to the reliability data in two prior TKA publications, the present study reported at least comparable performance. Miura et al. found almost perfect reliability intra- and inter-operator in the femoral coronal plane (ICC of 0.81 and 0.89, respectively), but only moderate to substantial reliability in the femoral transverse plane (ICC of 0.46 and 0.62, respectively) [10]. In another work, Schoenmakers et al. reported almost perfect inter-operator reliability (ICC range from 0.92 to 1.00) for six out of twelve degrees-of-freedom, including the femur along all axes and in the transverse plane, and the tibia in the transverse and sagittal planes [11]. These authors also reported almost perfect intra-operator reliability (ICC range from 0.90 to 1.00) for seven out of twelve degrees-of-freedom, including the femur along all axes and in the transverse plane, and the tibia along the proximal-distal axis and in the transverse and sagittal planes. Comparing the reliability in the three studies reveals some disparities, which could be caused by several factors, including the software, the assistance in implant placements, the imaging modality, the operator, and even the method of assessment. Additional research is thus necessary to fully understand the underlying factors contributing to the differences among studies and manage them at best.

For a better perception of the implications of the planning reliability, it is interesting to mention that the robotic system coupled to the planning software assessed in this study has been reported to cut the bones with root-mean-square errors below 1.8 mm or 1° in the six degrees-of-freedom for both the femur and the tibia [22]. Consequently, the reliability of the implant placements appeared to be adequate with regard to the accuracy of the bone cuts.

This study has some limitations. First, until further research with other types of implants and 3D preoperative planning software has been conducted, the generalizability of the findings remains unknown. Additional research will also be needed to evaluate the impact of the CT scan and the pre-planning of the reliability. Second, it happened that the surgeons had to plan the surgery with implants they thought were not of the ideal size, which could have contributed to lower the reliability. In this regard, it is important to mention that less than a third of the plannings were done with an implant size differing from what the surgeons would have selected and never with an implant differing by more than one size.

## Conclusion

This study reported high intra- and inter-operator levels of reliability for implant size selection and implant placements in a majority of the degrees-of-freedom. The lower reliability in implant placements observed in four degrees-of-freedom suggested both variations in the understanding of the planning objectives and a lack of specific definitions for the placements of the implants. This encourages further works to propose more precise means to describe the objectives of the surgical planning as well as the consideration of additional features in planning software to assist the operators, particularly in the four degrees-of-freedom with lower reliability. This being said, it should be noted that the maximum SEM of 1.3 mm or 1.7° remained relatively small and that further research is needed to determine the impact of the planning reliability on the clinical outcomes.

## Data Availability

Not applicable.
